# Anti-cancer activities of allyl isothiocyanate and its conjugated silicon quantum dots

**DOI:** 10.1038/s41598-018-19353-7

**Published:** 2018-01-18

**Authors:** Peng Liu, Mehrnaz Behray, Qi Wang, Wei Wang, Zhigang Zhou, Yimin Chao, Yongping Bao

**Affiliations:** 1 Norwich Medical School, University of East Anglia, Norwich, Norfolk United Kingdom; 2 School of Chemistry, University of East Anglia, Norwich, Norfolk United Kingdom

## Abstract

Allyl isothiocyanate (AITC), a dietary phytochemical in some cruciferous vegetables, exhibits promising anticancer activities in many cancer models. However, previous data showed AITC to have a biphasic effect on cell viability, DNA damage and migration in human hepatoma HepG2 cells. Moreover, in a 3D co-culture of HUVEC with pericytes, it inhibited tube formation at high doses but promoted this at low doses, which confirmed its biphasic effect on angiogenesis. siRNA knockdown of Nrf2 and glutathione inhibition abolished the stimulation effect of AITC on cell migration and DNA damage. The biological activity of a novel AITC-conjugated silicon quantum dots (AITC-SiQDs) has been investigated for the first time. AITC-SiQDs showed similar anti-cancer properties to AITC at high doses while avoiding the low doses stimulation effect. In addition, AITC-SiQDs showed a lower and long-lasting activation of Nrf2 translocation into nucleus which correlated with their levels of cellular uptake, as detected by the intrinsic fluorescence of SiQDs. ROS production could be one of the mechanisms behind the anti-cancer effect of AITC-SiQDs. These data provide novel insights into the biphasic effect of AITC and highlight the application of nanotechnology to optimize the therapeutic potential of dietary isothiocyanates in cancer treatment.

## Introduction

Allyl isothiocyanate (AITC) is produced by the hydrolysis of its glucosinolate precursor, sinigrin, which can be found in many commonly consumed cruciferous vegetables and is particularly abundant in mustard, horseradish and wasabi where it is responsible for the pungent taste^1^. Because of the pungent flavor, AITC is also used as a food additive known as mustard oil. AITC has been shown to possess a broad spectrum of anticancer activities in both cultured cancer cell lines and animal tumor models^1–3^. The mode of action for the chemopreventive activity of AITC is attributed primarily to the detoxification of carcinogens through activation of nuclear factor erythroid-related factor2 (Nrf2)^4^. AITC also inhibited the growth of various human cancer cell lines such as colorectal carcinoma^5^, lung cancer^6^, leukemia^7^, breast adenocarcinoma^8^, bladder cancer^3,9^, neuroblastoma^10^, hepatoma^11^ and prostate cancer cells^2,12^. The mechanisms are likely to involve DNA damage^6^, cell cycle arrest and apoptosis^8,12,13^ and binding to thiol-reactive groups of several cellular targets such as DNA topoisomerase 2, p53 and tubulins^4,14,15^. In addition, AITC has been reported to suppress metastasis via inhibition of invasion and migration^3,11^ in neoplastic cells. The antiangiogenic activity of AITC has also reported in *in vivo* studies^16,17^. However, findings from epidemiological studies on the association between cruciferous vegetable intake and cancer risk are generally inconsistent^18,19^. The hormetic effects of isothiocyanates may be the cause of the complex biological impact of a cruciferous vegetable diet^19^.

In toxicology, hormesis refers to a dose–response relationship with a stimulatory response at low doses and an inhibitory response at high doses^20^. Many drugs have been found to demonstrate such contradictory effect at high and low doses in the same individual. This reaction, also referred to as a ‘biphasic dose response’, has shown significance in establishing the modality of a drug. On the other hand, mild stress stimuli can often trigger an adaptive stress response in order to maintain homeostasis, so that whilst a high dose of an insult brings harm, a low dose could promote health^21^. Dietary phytochemicals have been reported to be prominent hormetic stressors that affect various signaling pathways associated with the progression of diverse diseases, especially cancers^19,22–24^. Isothiocyanates for example, have been reported to kill cancer cells at high doses but to promote cancer cell proliferation and survival at low doses^19,22,23^. It is thus crucial to optimize the beneficial effects and minimize the potential risks of isothiocyanates in cancer prevention and treatment.

Over the last decade, the growth of nanotechnology has opened several new vistas in medical sciences, especially in the field of cancer treatment. Amongst the various types of nanomaterials, semiconductor nanoparticles (NPs), also referred to as quantum dots (QDs), have been developed for both bio-imaging and therapeutic purposes because of their unique electronic and optical properties. Within the family of QDs, silicon quantum dots (SiQDs) have been preferred in biomedicine applications because of their low inherent toxicity, in contrast to all II-VI types of QDs based on heavy metals such as cadmium, lead and arsenic^25^. The photoluminescent properties of SiQDs are as a result of quantum confinement effect while their physiochemical properties largely depend on the surface reconstruction and termination^25,26^. Further exploration of the surface functionalization of SiQDs may facilitate their application as drug carriers for chemotherapeutic agents, photosensitizers, siRNA and gene therapeutic agents and can also act as multifunctional entities for both imaging and therapy at the same time^26–28^.

Here for the first time, the design of a new multifunctional NP system with SiQDs as carrier and AITC as surface ligand is reported. The objectives of the present study are to investigate the differences between the bioactivities of AITC and AITC-SiQDs, and to examine the potential mechanisms/application of AITC-SiQDs to act as multifunctional vehicle for cancer therapy.

## Materials and Methods

### Materials

AITC, Methylthiazolyldiphenyl-tetrazolium bromide (MTT), dimethyl sulfoxide (DMSO), DL-Buthionine-sulfoximine (BSO), N-acetyl-L-cysteine (NAC) and Bradford reagent were all purchased from Sigma-Aldrich. Complete protease inhibitors were obtained from Roche Applied Science. Primary antibodies to Nrf2 (Catalog No. 13032), Sam68 (Catalog No. 333), Ku70 (Catalog No. 1486), β-actin (Catalog No. 7210), HRP-conjugated goat anti-rabbit and rabbit anti-goat IgG were all purchased from Santa Cruz Biotechnology. Anti-human CD31 (Catalog No. 555444) was purchased from BD Biosciences. Secondary antibodies conjugated with Cy3 were obtained from Jackson Immuno Research. Nrf2 siRNA was obtained from Applied Biosystems (Sense strand: 5′-CCUUAUAUCUCGAAGUUUUtt-3′; antisense strand: 5′-AAAACUUCGAGAUAAGGtg-3′). AllStars negative control and HiPerFect transfection reagent were purchased from Qiagen. LysoTracker® Red DND-99 was obtained from ThermoFisher.

### Synthesis and characterization of AITC-SiQDs

Hydrogen terminated SiQDs were synthesized by galvanostatic anodization of porous silicon layer as reported previously^29^. Bromine-functionalised SiQDs were synthesised by reacting the hydrogen terminated SiQDs with allyl bromide. The resultant product was dried under vacuum and reacted with potassium thiocyanate to form isothiocyanate-functionalised SiQDs, i.e. AITC-SiQDs (Supplementary Fig. S1). The elemental composition of the products was confirmed by X-ray photoelectron spectroscopy (XPS) (Supplementary Fig. S2). The optical properties of AITC-SiQDs were examined by UV−vis absorption (UV−vis) and photoluminescence (PL) spectroscopy (Supplementary Fig. S3). Thermal Gravimetric Analysis (TGA) was used to estimate the quantity of ligands on the surface of SiQDs (Supplementary Fig. S4). The sizes of AITC-SiQDs were measured by Transmission Electron Microscopy (TEM) (Supplementary Fig. S5). The hydrodynamic diameter of AITC-SiQDs was measured by Dynamic Light Scattering (DLS) within different environment (Supplementary Table S1).

### Cell culture

Human umbilical vein endothelial cells (HUVECs) were obtained from TCS Cellworks and murine MII perivascular cells (M2) were isolated as previously described^30^. HHL5, the immortalized human hepatocyte-derived line 5, was provided by Professor Arvind Patel, Medical Research Council Virology Unit, UK^31^. All other cell lines were purchased from ATCC. HUVECs were cultured in Endothelial Cell Growth Medium 2 (PromoCell) supplemented with antibiotics (penicillin (100 U/ml) and streptomycin (100 μg/ml) at 37 °C, 5% (v/v) CO_2_. All other cell lines were routinely cultured in Dulbecco’s modified Eagle’s medium (DMEM) supplemented with foetal bovine serum (10%), 2 mM glutamine, penicillin (100 U/ml) and streptomycin (100 mg/ml) at 37 °C, 5% (v/v) CO_2_. HUVECs were used between the fifth and ninth passages and M2 were used between passages 35 and 40 for all experiments. For these two cell lines, HUVECs and M2 cells were grown in flasks coated with 10 µg/ml type-I collagen (Sigma).

### Cell viability assay

The cell viability 3-[4,5-dimethylthiazol-2-yl]-2, 5 diphenyl tetrazolium bromide (MTT) assay was employed to determine the toxicity of AITC or AITC-SiQDs towards cultured cells. Cells were seeded in 96-well plates at a concentration of 0.5–1.0 × 10^4^ cells in a final volume of 100 μl per well. When cells were at approximately 70–80% confluence, different doses of AITC or AITC-SiQDs treatments were added with fresh medium, DMSO (0.1%) used as controls. After 24 hours, the medium was removed, 100 μl (5 mg/ml) MTT added, and the mixture incubated at 37 °C for 1 hour to allow metabolism of MTT. The formazan formed was then re-suspended in 100 μl DMSO per well. The final absorbance was recorded using a microplate reader (BMG Labtech Ltd) at a wavelength of 550 nm and a reference wavelength of 650 nm. The IC50 values were determined using the CalcuSyn Software (Biosoft, Cambridge).

### Alkaline Comet assay

The alkaline Comet Assay, also called single-cell gel electrophoresis, is a sensitive and rapid technique for quantifying DNA damage in individual cells. HepG2 cells were seeded in 24-well plates and allowed to grow to 70–80% confluence, then placed in experimental conditions. Cells were then harvested and resuspended in PBS containing 10% DMSO and frozen at −80 °C until the alkaline comet assay was performed as described previously^32^. For each sample, 100–200 comets were randomly analyzed with images captured by fluorescence microscopy (Axioplan2, Carl Zeiss) and scored using Comet Assay IV Lite analysis software (Perceptive Instruments, Bury St Edmunds). DNA damage was expressed as tail intensity (% DNA in the comet tail) for statistical analysis because it has a linear relationship to DNA break frequency, is relatively unaffected by threshold settings and yields the widest possible range (i.e., 0–100%)^33^.

### Wound assay

Cells were seeded in 24-well plates at 2 × 10^5^ cells/ml. After cells reached 100% confluence, scratches were made with a 1 ml pipette tip across the center of the wells without changing the medium. Detached cells were removed by gently washing twice with medium. The wells were then filled with fresh medium containing different treatments with DMSO (0.1%) as control. Each treatment was performed at least in triplicate. Cells were grown for a further 48 hours then washed twice with PBS, fixed with ice-cold methanol for 10 minutes, and stained with 1% crystal violet for 30 minutes. At least 3 pictures were taken within each well of the stained monolayer on an inverted microscope at 5x magnification. The wound area was quantitatively evaluated using ImageJ^34^, at least 10 pictures were used in each treatment. Cell migration was calculated as follows: Migration % = 1 − (Area W − Area C)/AreaC %, where Area W is the wound area from treated wells and Area C is the wound area from the control wells.

### Tube formation in a 3D model

HUVEC and M2 were co-cultured in collagen type I gel as described previously^30^. Different doses of treatment were added to the medium (top of 3-D collagen gel) with DMSO (0.1%) as control, the medium was being changed every 48 hours. At day 5, whole-mount immunohistochemistry of the 3D collagen cultures was performed for CD31 and counterstained with DAPI. Samples were examined by fluorescence microscopy (Axioplan2, Carl Zeiss). In five random fields from each sample, the total lengths of CD31-positive tube-like structures were measured by Volocity 4.0 (Improvision). Cumulative tube lengths *per* area are expressed as mm/mm^2^.

### Protein extraction and Western blot analysis

For total protein, cells were washed twice with ice-cold PBS, incubated in 20 mM Tris-HCl (pH 8), 150 mM NaCl, 2 mM EDTA, 10% glycerol, 1% Nonidet P40 (NP-40) containing complete proteinase inhibitor for 30 minutes at 4 °C and then harvested and centrifuged at 13,600 g for 15 minutes at 4 °C. Supernatant was collected and the protein concentration determined by the Brilliant Blue G dye-binding assay of Bradford using bovine serum albumin as a standard. For the nuclear protein, the extraction was performed using a Nuclear Extract Kit (Active Motif), following the manufacturer’s instructions.

Protein extracts were heated at 95 °C for 5 minutes in loading buffer and loaded onto 10% SDS-polyacrylamide gels together with a molecular weight marker. After routine electrophoresis and transfer, the PVDF membrane was blocked with 5% fat-free milk in PBST (0.01% Tween 20) for 1 hour and incubated with a specific primary antibody overnight at 4 °C. The membrane was washed three times for 5 minutes with PBST and then incubated with the secondary antibody for 1 hour. After further washing, antibody binding was determined by a chemiluminescence detection kit (Amersham, GE Healthcare) and densitometry was measured by Fluor Chem Imager (Alpha Innotech).

### Knockdown Nrf2 by siRNA

HepG2 cells were seeded in 24-well plates at density of 1–1.5 × 10^5^ cells in a volume of 0.5 ml per well, then transfected with siRNA for Nrf2 or Allstars (that has no homology to any known mammalian gene) for 24 hours following the manufacturer’s instructions. Wound assay/comet assay were then performed as above. The siRNA knockdown efficiency of Nrf2 was characterized using Western blot analysis (Supplementary Fig. S6).

### Reactive oxygen species measurement

The production of intracellular reactive oxygen species (ROS) was measured using the chloromethyl derivative of the fluorescent probe 2′,7′-dichlorodihydrofluorescein diacetate (CM-H_2_DCFDA) (Invitrogen). To evaluate AITC-SiQDs induced ROS, cells were seeded in 6-well plates and treated when they reached 70% confluence with 20 μM AITC-SiQDs for 1, 3, 6, 12 and 24 hours. The wells were then washed with PBS and incubated with 5 μM CM-H_2_DCFDA for 30 minutes at 37 °C. Subsequently, the cells were collected, centrifuged and re-suspended in 0.8 ml PBS. Intracellular ROS production was determined by detection of fluorescent intensity of the oxidized product DCF in the FL1-A channel with a flow cytometer (Cube 6, Sysmex Partec).

### Confocal laser scanning microscopy (CLSM)

HepG2 were plated onto 10 mm glass coverslips in 24-well plates at a concentration of 2 × 10^5^ cells/ml and incubated for 48 hours at 37 °C, 5% CO_2_. Cells were then treated with 50 µM AITC-SiQDs (excitation/emission: 350/440), or 0.1% DMSO as control for 1, 3, 6, 12, 24 hours. After exposure, cells were washed twice with PBS and fixed with 4% paraformaldehyde for 10 minutes. Cover slips with cells were inverted and mounted on a microscope slide. For lysosome staining, during the last 10 minutes of AITC-SiQDs treatment, cells were exposed to 1 µM LysoTracker (excitation/emission: 577/590). The cells were then washed with fresh medium for 10 minutes at 37 °C, 5% CO_2_, washed then fixed as above. CLSM was performed on a Zeiss LSM510 META confocal microscope using a 10x objective lens for imaging. Laser beams with 364, 488 and 543 nm excitation wavelengths were used to image AITC-SiQDs, bright field and lysosome respectively.

### Statistics

Data are represented as the mean ± SD. The differences between the groups were examined using the one-way ANOVA test, or Student’s t-test. A p value <0.05 was considered statistically significant.

## Results

### Biphasic effect of AITC on cell viability, DNA integrity, migration and angiogenesis

**Figure 1:**
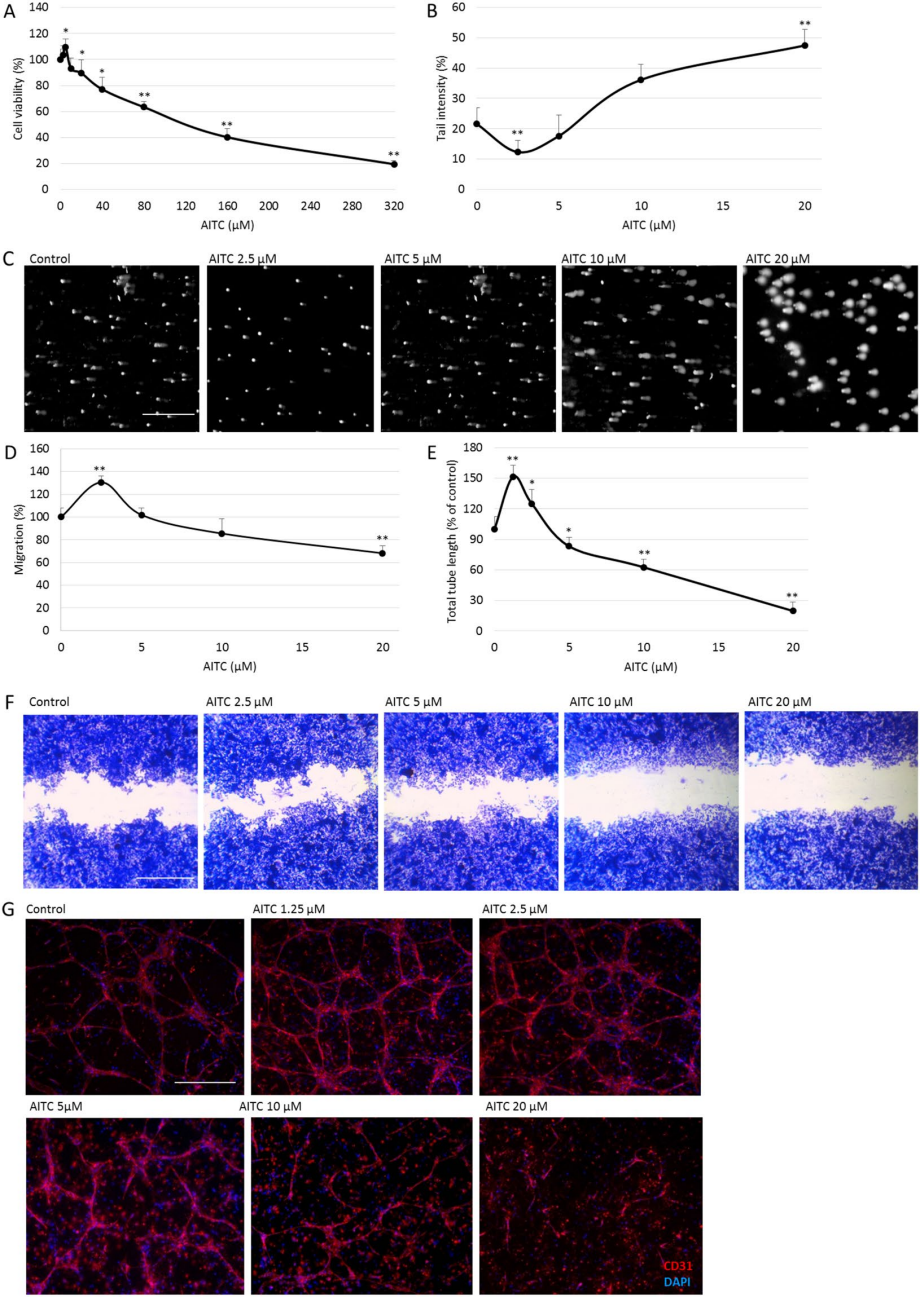
Effect of AITC on cell viability, DNA integrity, migration and angiogenesis. (**A**) HepG2 cell viability at 24 hours AITC treatment was determined by MTT assay. Data are presented as mean ± SD (n ≥ 5), *p < 0.05, **p < 0.01 compared to control. (**B**) HepG2 DNA damage at 24 hours AITC treatment was detected by the Comet assay. Data are presented as mean ± SD (n ≥ 5), **p < 0.01 compared to control. (**C**) Representative pictures from the comet assay. Scale bar = 500 µm. (**D**) HepG2 cell migration at 48 hours AITC treatment was measured by the wound assay. Data are presented as mean ± SD (n ≥ 5), **p < 0.01 compared to control. (**E**) Effect of AITC on tube formation of HUVECs in a 3D co-culture with pericytes model. The total lengths of CD31 positive tubes were measured and expressed as mean ± SD (n ≥ 5), *p < 0.05, **p < 0.01 compared to control. (**F**) Representative phase contrast images from the wound assay. Scale bar = 1 mm. (**G**) Representative pictures from the immunostaining of CD31 (red) and DAPI (blue), scale bar = 500 µm.

The antineoplastic activity and genotoxicity of AITC were measured using MTT and Comet assays respectively. Results indicated that AITC decreased the metabolic activity of HepG2 cells in a dose-dependent manner after 24 hours. When cells were treated with AITC 40–320 µM, cellular viability was significantly inhibited (77.1–19.4% compared to control); however, a significant stimulation of cell viability was found with 5 µM AITC (Fig. 1A).

The genotoxicity of AITC was also measured at non-cytotoxic concentrations (0–20 µM) using the Comet assay. The baseline DNA damage, represented as tail intensity percentage, in control cells was 21.57% and there was a significant increase at 10 and 20 μM AITC treatment after 24 hours, 36.12 and 47.48% respectively; while at 2.5 µM AITC decreased DNA damage to 12.3% (Fig. 1B,C). There have been several studies reporting the mechanism behind the DNA damage caused by AITC^6,35–37^. Here, the induction of DNA damage with the higher dose of AITC was accompanied by downregulation of DNA repair protein Ku70 (Supplementary Fig. S7).

To assess whether AITC also affects cell migration, which is an indication of epithelial–mesenchymal transition and the aggressive phenotype of malignant tumor, a wound assay was performed to measure the cell migration under different doses of AITC treatment after 48 hours. Around 30% inhibition of migration compared to the control was observed with 20 μM AITC exposure. Again, a significant increase of cell migration was observed with a low dose (2.5 μM) of AITC (130% compared to control) (Fig. 1D and F).

Angiogenesis, a process leading to the formation of new blood vessels, is required for both cancer progression and metastasis^38^. Therefore, the effect of AITC on the ability of HUVECs to form capillary-like tubular structures was tested in a 3D co-culture model with M2. Mature tube formation, clearly observed in the control, was significantly disrupted with AITC treatment (>5 μM), as illustrated by a sharp decrease in formed total tube length. However, at lower doses AITC (1.25 and 2.5 μM) significantly promoted the formation of capillary tubular structures (Fig. 1E and G).

These data suggest that there was a biphasic dose response from AITC treatment in all tested endpoints. Low dose AITC could be beneficial to patients with cardiovascular disease because of its stimulatory effect on endothelial cell tube formation. Conversely, for cancer treatment only high doses of AITC should be used, as low doses stimulated cancer cell viability and migration, and also restored genomic stability and promoted angiogenesis.

### Low dose AITC stimulation effect is mediated by Nrf2/GSH signaling

**Figure 2:**
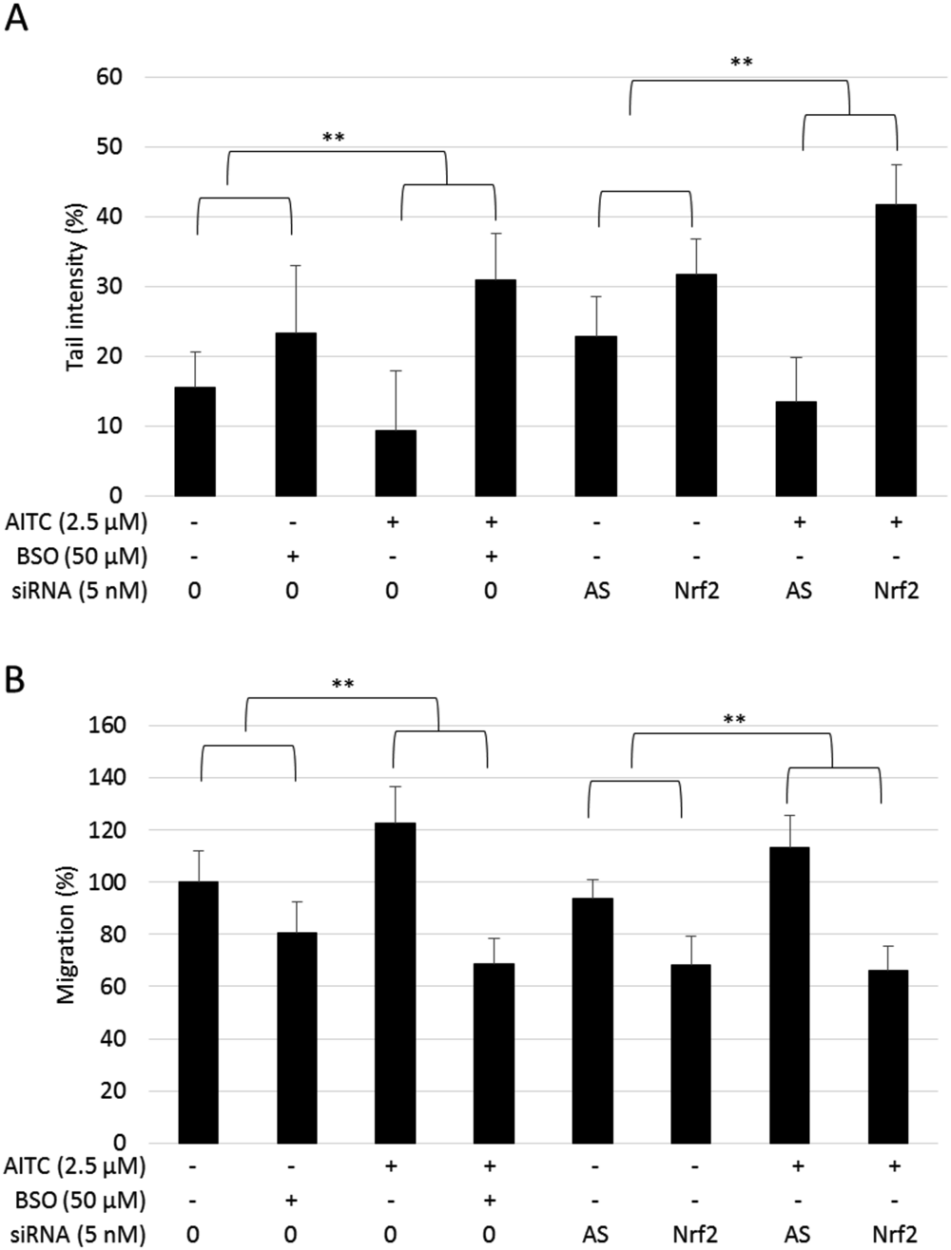
Effect of Nrf2 siRNA and GSH inhibition on HepG2 cell DNA damage and migration exposed to AITC. Allstars (AS) was used as a control for knockdown. Cells were incubated with 2.5 μM AITC or DMSO (0.1%) control with/without 50 μM BSO co-treatment. (**A**) DNA damage was measured by the Comet assay after 24 hours treatments. (**B**) Cell migration was measured by the Wound assay after 48 hours. Data are presented as mean ± SD (n ≥ 3), **p < 0.01 between the indicated groups (t-test).

A low dose stimulation effect could be a risk factor in the role of AITC in cancer prevention and treatment. Isothiocyanates have been shown to activate Nrf2, a master transcription factor involved in cell redox homeostasis, stress adaptation and cytoprotection^39^. The role of Nrf2 in the low dose stimulation effect of AITC on DNA damage and cell migration was investigated using a siRNA knockdown approach. As shown in Fig. 2, Nrf2 knockdown cells showed significantly increased DNA damage and reduced cell migration compared to non-transfected control cells. This suggested Nrf2 is involved in HepG2 cell genomic stability and migration. AITC at 2.5 µM reduced DNA damage and promoted cell migration in HepG2 cells. Cells transfected with Allstar negative control showed similar effects with AITC treatment to the non-transfected ones. In contrast, these stimulatory effects from 2.5 µM AITC were abolished upon Nrf2 knockdown, i.e. DNA damage increased from 9.4% to 41.8%; cell migration decreased from 122.7 to 66.1% (p < 0.01). These data strongly indicated that Nrf2 was involved in the low dose stimulation effect of AITC in DNA damage and cell migration.

Glutathione (GSH) is the most abundant non-enzymatic antioxidant molecule in the cell and is essential for redox regulation. One of the Nrf2 target genes, γ-glutamyl cysteine synthetase (γ-GCS), is the rate limiting enzyme of GSH synthesis^39^. Therefore, the involvement of GSH in the AITC stimulation effect was studied using buthionine sulfoximine (BSO), a specific inhibitor of γ-GCS. The inhibition efficiency of BSO in the intracellular GSH level was characterized using HPLC (Supplementary Fig. S8). BSO increased DNA damage by 1.5-fold and decreased cell migration by approximately 20% compared with the control. Co-treated with BSO, 2.5 µM AITC treatment showed no stimulatory effect on DNA damage or cell migration (Fig. 2). Therefore, it can be concluded that the Nrf2/GSH signaling pathway plays an essential role in low dose AITC inhibition of genomic instability and stimulation of cell migration.

### AITC-SiQDs abolished the low dose stimulation effect of AITC

**Figure 3:**
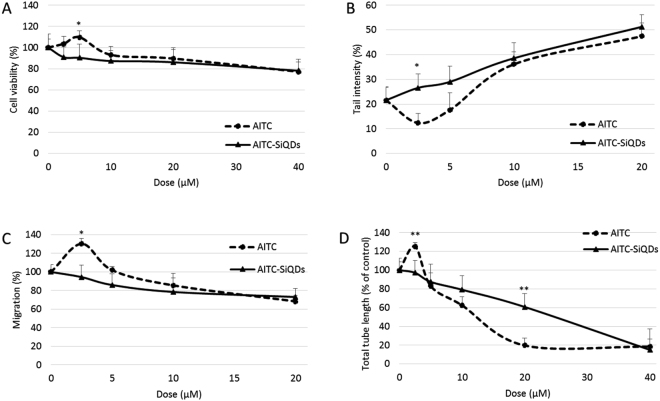
Effect of AITC-SiQDs was compared with AITC on HepG2 cell viability (**A**), DNA integrity (**B**), migration (**C**) and tube formation in the 3D HUVEC co-culture with the pericytes model (**D**). Data are presented as mean ± SD (n ≥ 3), *p < 0.05, **p < 0.01 compared to corresponding AITC treatment.

The effect of AITC-SiQDs on cell viability was initially screened using the MTT assay. Cells were incubated with different concentrations of AITC-SiQDs for 24 hours with AITC as the positive control for 24 hours. As shown in Fig. 3A, there was no significant difference between the cytotoxicity of AITC-SiQDs and AITC at high dose (nearly 20% decrease from 40 µM treatment); but there was a significant difference on the cell viability between low dose AITC-SiQDs and AITC treatments, thus cell viability was approximately 90% compared to control with 2.5 and 5 µM of AITC-SiQDs treatment while AITC treatment increased cell viability to 103–110% of the control.

To confirm the cytotoxicity of AITC-SiQDs came from the surface ligand instead of the SiQDs core, amine-capped SiQDs (NH_2_-SiQDs)^40^ were used as negative control in the MTT assay. Results in Supplementary Fig. S9A showed that AITC-SiQDs decreased the viability of HepG2 cells after 24 hours incubation, in contrast, NH_2_-SiQDs showed no significant cytotoxicity. Moreover, the cytotoxicity of AITC-SiQDs was measured in HHL5 cells to test their effect on normal cells. Results showed that there were no significant difference between the cytotoxicity from AITC and AITC-SiQDs in HHL5 cells (Supplementary Fig. S9B).

The effect of AITC-SiQDs on DNA damage, cell migration and angiogenesis was also examined **(**Fig. 3B–D**)**. At 20 µM AITC-SiQDs induced DNA damage (2.5-fold increase compared to control); inhibited cell migration (60% decrease compared to control); and inhibited tube formation in the 3D co-culture model (60% decrease compared to control). More importantly, AITC-SiQDs at 2.5 µM showed no stimulation in contrast to AITC.

### Effect of AITC/AITC-SiQDs on the nuclear accumulation of Nrf2

**Figure 4:**
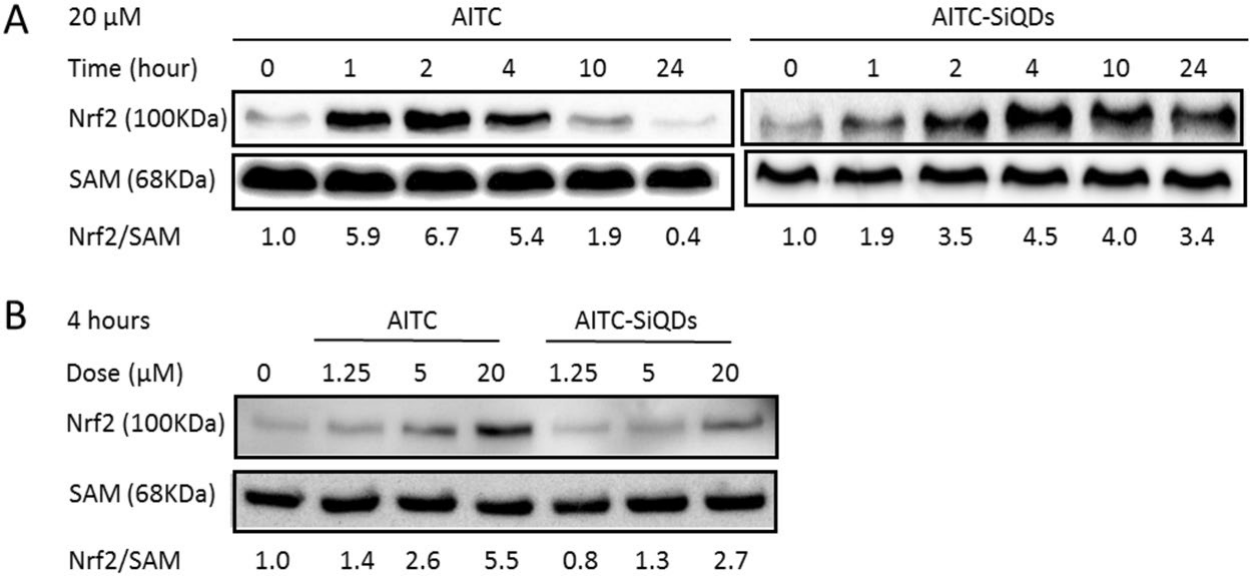
Effect of AITC or AITC-SiQDs on Nrf2 nuclear accumulation in HepG2 cells. Nrf2 was detected by Western blotting and quantified against SAM as a loading control, results were expressed as fold induction relative to controls. (**A**) Time course of the effect of 20 µM AITC or AITC-SiQDs on Nrf2 nuclear protein level. (**B**) Dose response of AITC or AITC-SiQDs at 4 hours on Nrf2 nuclear protein level.

The involvement of AITC-SiQDs in the activation of Nrf2 compared to AITC in HepG2 cells was examined. Nuclear protein was extracted and Nrf2 measured by Western blotting. As shown in Fig. 4, untreated cancer cells had low Nrf2 levels in the nucleus consistent with the continuous degradation of Nrf2 under homeostasis. With 20 µM AITC treatment, a significant increase of Nrf2 protein in the nucleus was observed after 1 hour but this started to decrease after 4 hours, while with the same dose AITC-SiQDs treatment caused the nuclear protein level of Nrf2 to increase compared to control for at least 24 hours. In addition, AITC treatment (1.25, 5, 20 µM) for 4 hours induced a significant and dose-dependent increase of Nrf2 in the nucleus, but AITC-SiQDs treatment showed much milder effect in this regard compared to the same dose of AITC. These data indicated different dynamics of Nrf2 activation between AITC and AITC-SiQDs.

### Cellular uptake of AITC-SiQDs

**Figure 5:**
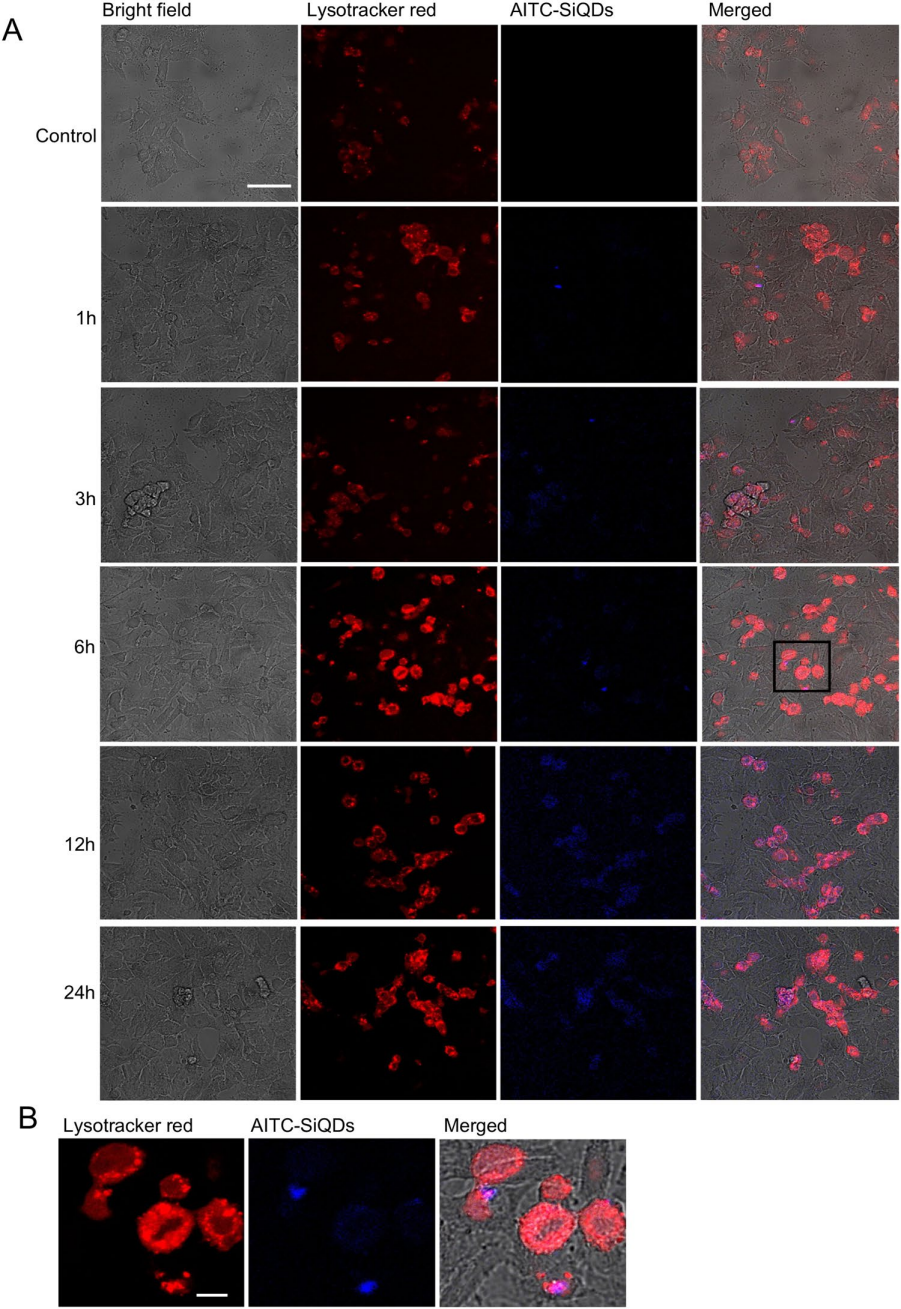
Confocal imaging and quantification of AITC-SiQDs cellular uptake in HepG2 over 24 hours. (**A**) Representative images of the time course of AITC-SiQDs signalling in HepG2 cells. The red channel shows fluorescence of Lysotracker and the blue channel the fluorescence of AITC-SiQDs, a merged image of red, blue and bright channels is shown in the final column. Scale bar = 50 µm. (**B**) High resolution images from 6 hours of AITC-SiQDs localization into the lysosomes of HepG2 cells, area indicated by the black rectangle in the merged images. Scale bar = 10 µm.

The nature of the internalization of NPs with cells is a key factor in their bioactivity. To investigate the cellular uptake of AITC-SiQDs, HepG2 cells were incubated with fluorescent AITC-SiQDs and analyzed by CLSM; additional studies were performed using Lysotracker red, a fluorescent cell-permeant acidic organelle-selective marker. The Lysotracker probes, which comprise a fluorophore linked with a weak base that is only partially protonated at neutral pH, freely penetrate cell membranes and are typically used to mark organelles including lysosomes and some late endosome at acidic pH^41^. The confocal microscopy images are shown in Fig. 5. The control cells (treated with 0.1% DMSO) did not exhibit fluorescence. After 1 hour of incubation with AITC-SiQDs, a blue fluorescence signal was observed inside the cells; this peaked around 12 hours indicating the internalization of a large number of AITC-SiQDs. At 24 hours, there were still signals from internalized AITC-SiQDs which indicated the excretion of QDs took at least this length of time. Lysosomes were identifiable within HepG2 cells, and there was a clear co-localization of AITC-SiQDs within lysosomal structures at all time points investigated (Fig. 5B), which indicated that QDs were taken up by the cells through endocytosis as is the case for most types of NPs^42^.

### Anti-cancer properties of AITC-SiQDs is mediated by ROS

To check that the observed effect of AITC-SiQDs on abolishing the low dose stimulation effect was not specific to HepG2, the effect of AITC-SiQDs on colorectal adenocarcinoma cells, Caco-2, was investigated. Caco-2 cells were more sensitive towards AITC than HepG2 cells. As anticipated, results showed 80 µM AITC-SiQDs decreased cell viability to 31.77% compared to control in Caco-2 while only 78.19% in HepG2 cells (Supplementary Fig. S10A). As presented in Supplementary Fig. S10B,C, AITC-SiQDs abolished the stimulation effect seen with AITC (2.5 and 5 µM) on cell migration; and the prolonged induction of Nrf2 nuclear accumulation by AITC-SiQDs compared to the sharp induction by AITC was also observed in Caco-2. These results suggest that this effect of AITC-SiQDs is not cell line specific.

**Figure 6:**
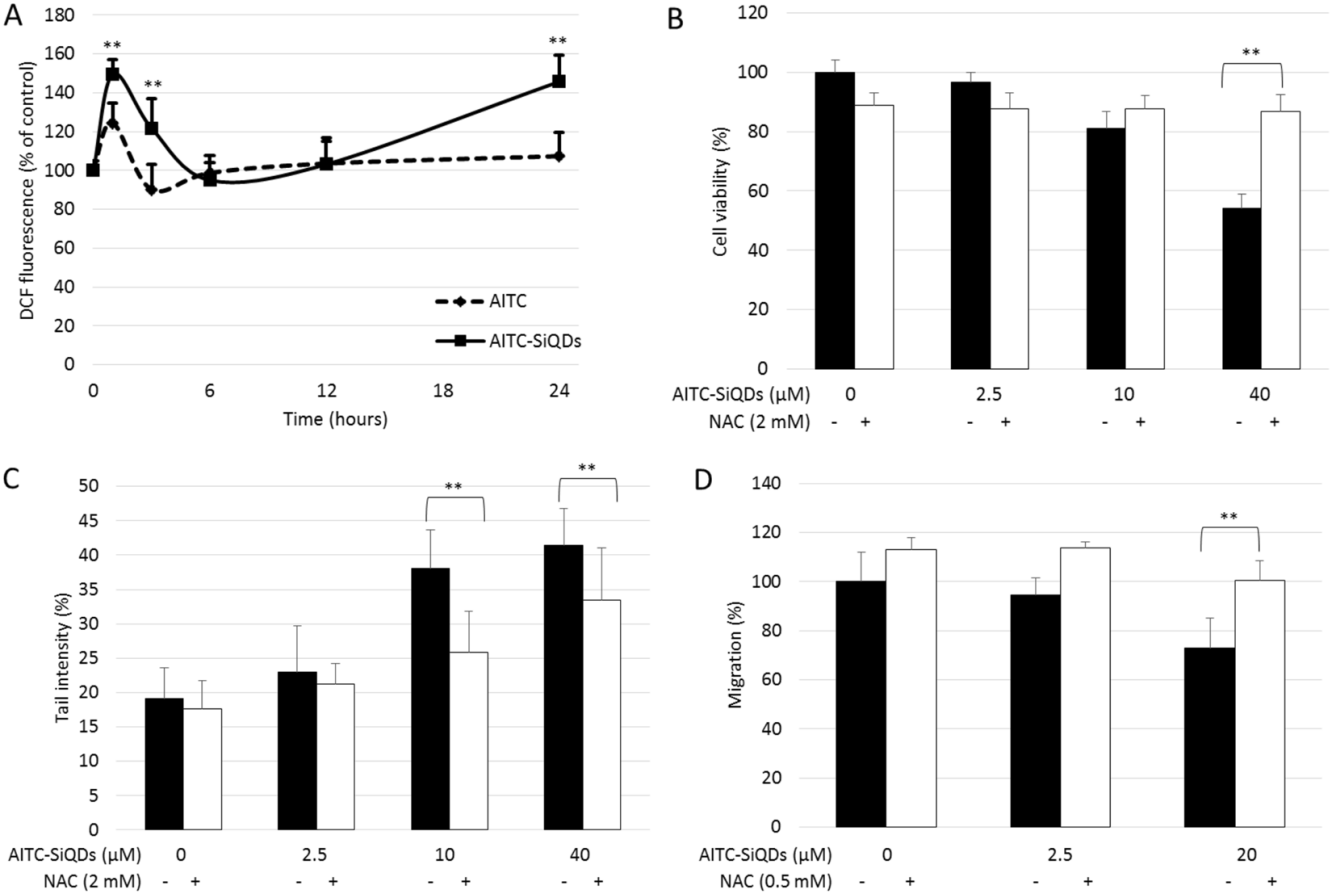
Anti-cancer properties of AITC-SiQDs is mediated by ROS. (**A**) Caco-2 cells were incubated with 20 µM AITC or AITC-SiQDs over 24 hours with DMSO (0.1%) as control, ROS was measured using flow cytometry. Data are presented as mean ± SD (n ≥ 3), **p < 0.01 compared to corresponding AITC treatment. (**B**) Caco-2 cells were incubated with different doses of AITC-SiQDs with or without NAC (2 mM) for 24 hours. Cell viability was measured by the MTT assay. (**C**) HepG2 cells were incubated with different doses of AITC-SiQDs with or without NAC (2 mM) for 24 hours. DNA damage was measured by the Comet assay. (**D**) HepG2 cells were incubated with different doses of AITC-SiQDs with or without 0.5 mM NAC for 48 hours. Cell migration was measured by the wound assay. Data are presented as mean ± SD (n ≥ 5), **p < 0.01 between the indicated groups (t-test).

ROS generation is one of the common mechanisms by which NPs exert toxicity; accordingly the intracellular ROS was measured using a H2DCFDA probe, a stable nonpolar dye that diffuses readily into cells and yields DCFH. Intracellular ROS, in the presence of peroxidase, converts DCFH to fluorescent DCF. As shown in Fig. 6A, 20 µM AITC or AITC-SiQDs treatment both caused significant increase of ROS at 1 hours in Caco-2 cells, i.e., the DCF fluorescence intensity measured as 124.6% and 149.6% of the control from AITC and AITC-SiQDs respectively. ROS returned to control level after 3 hour in AITC-treated cells, but in AITC-SiQDs treated cells DCF intensity was 121.8% and 145.7% of control at 3 and 24 hours. To exam further whether the AITC-SiQDs induced anti-proliferative response is related to ROS, a well-known antioxidant N-acetyl-L-cysteine (NAC) was introduced to quench ROS production. Results from their co-treatment showed that NAC completely blocked the reduction in cell viability caused by AITC-SiQDs in Caco-2 (Fig. 6B), this suggested that ROS generated by AITC-SiQDs participated in the anti-proliferation effect. Further results indicated that co-treatment with NAC reduced DNA damage caused by AITC-SiQDs and impaired the inhibitory effect of AITC-SiQDs on cell migration (Fig. 6C,D). Taken together, these data suggest a ROS mediated mechanism behind the bioactivities of AITC-SiQDs.

## Discussion

Phytochemicals can play an important role in cancer prevention and treatment. Some phytochemicals exert health benefits by inducing adaptive cellular stress responses. Indeed, recent findings suggest that several phytochemicals exhibit biphasic dose responses on cells with low doses activating signaling pathways that result in increased expression of genes encoding cytoprotective proteins such as antioxidant enzymes, protein chaperones, growth factors and mitochondrial proteins^22^. One example is the isothiocyanates from cruciferous vegetables and their activation of Nrf2.

A common mechanism behind the chemopreventive activity of isothiocyanates is the activation of Nrf2, which lead to the induction of iron metabolism proteins, phase II detoxifying enzymes, phase III transporters and antioxidant proteins. Among these antioxidants, GSH synthesis and utilization are regulated by Nrf2^43^. However recent studies have demonstrated a negative aspect of Nrf2 in cancer. Several genes activated by Nrf2 are associated with cancer progression, such as those regulating proliferative signaling and reprograming energy metabolism^44,45^. Nrf2 has also been reported to be a major driver of hepatocarcinogenesis^46^, and to be constitutively activated in many types of cancer cells or tumor samples from patients, which contributes to aggressive cancer phenotypes such as increased proliferation, metastasis and chemotherapeutic resistance. Overexpression of Nrf2 is associated with poor prognosis in cancer patients^45^. Recent applications of Nrf2 inhibitors and knockdown treatment have been reported to effectively enhance chemotherapy^44,47^. Therefore, there is a pressing need to define the boundaries between the positive and negative effects of Nrf2 in cancer, and to establish a precise rationale for undertaking Nrf2 therapeutic targeting.

The results reported here showed that AITC, a naturally occurring isothiocyanate, exhibited biphasic anti-cancer properties in HepG2 cells: high dose (≥20 µM) of AITC decreased cell viability, increased DNA damage and inhibited cell migration and angiogenesis; while low doses (1.25–2.5 µM) produced an opposite effect. One of the main mechanisms behind the low dose stimulation effect is linked to the induction of Nrf2 signaling by AITC as siRNA knockdown treatment abolished the stimulations. BSO co-treatment also significantly reduced the effects of low dose AITC indicating the involvement of GSH in the stimulatory effect. In this context it should be noted that the physiological concentration of AITC following by consumption of a meal rich in cruciferous vegetables or from supplements is around 1–5 µM in human plasma^1^, which means for most people the exposure of AITC would be within the subtoxic stimulatory dose range, which could be a risk factor for those who have transformed cells in the body.

It has been demonstrated here for the first time that by using nanotechnology the biphasic effect of AITC can be avoided. The biological and optical properties of AITC-SiQDs, combining the anti-cancer activity of AITC as the surface ligand and the photoluminescence of the SiQDs core, were both exploited in this nanoscale system. Results showed that at high doses AITC-SiQDs exhibited similar effects to that of AITC while at low doses were free from the stimulatory effect. Since the low dose AITC stimulation effect was mediated by Nrf2 signaling, the time- and dose- responses of Nrf2 signaling from AITC-SiQDs treatment were examined comparing to that from AITC. The accumulation of nuclear Nrf2 induced by AITC-SiQDs was found to be much less and to act over a longer time than that induced by AITC alone. Confocal imaging confirmed that the internalization and excretion of AITC-SiQDs took at least 24 hours, distinctively different from the free diffusion of AITC. Although the underlying mechanism is not yet fully understood, the different patterns of Nrf2 activation may be the key of AITC-SiQDs escaping the biphasic response shown by AITC, especially when at low doses the cytotoxic effects could be very limited. Further investigation is needed to confirm the differences between the bioactivities induced by a mild, long-lasting activation of Nrf2 and acute activation, such as the induction of detoxifying enzymes and antioxidant proteins, and the cross-talk with autophagy, metabolic activities, and other transcription factors such as NF-κB and p53.

Cellular uptake and excretion of NPs affect their biomedical applications. Endocytosis and exocytosis have been identified as major pathways for NPs entering and exiting the cells respectively. The endocytosed NPs are normally found in membrane bound organelles in the cytoplasm^42^, with fewer reported to be in the cytoplasm or other organelles such as nucleus^48^ and endoplasmic reticulum^49^. Cellular excretion of NPs is generally realized through lysosomal secretion, which last from 0.5 to 48 hours according to different reports^50^. The interaction between SiQDs aggregates and receptors in the endosome was found to be a determining factor for the removal process of SiQDs^51^. In this study, the luminescence from AITC-SiQDs were still observed in the lysosome after 24 hours incubation, indicating that the lysosome was still intact and that AITC-SiQDs had not been degraded. Therefore, AITC-SiQDs can be used to track and monitor *in vitro* over long timescales, and the longer retention of AITC-SiQDs in cells could contribute to the toxic effects especially at high doses. The role of ROS in the anti-cancer activities of AITC-SiQDs was also demonstrated by counteraction from the co-treatment of NAC. Both AITC and SiQDs have been reported to exert cytotoxicity via disrupting ROS homeostasis^4,52^. Prolonged intracellular retention of AITC-SiQDs may be better to exert their desired bioactivates, in this case, the cytotoxicity in comparison to AITC alone.

In summary, this nano delivery system presents an encouraging platform to avoid the biphasic effect of AITC administrated alone. Further *in vivo* studies must be performed to extrapolate the dose-effects found in the *in vitro* experiments. Together with other advantages that could be provided by this nanoscale delivery system, such as passive tumor targeting and real time monitoring^53^, AITC-SiQDs have the potential to be used in anti-cancer drug delivery.

## Electronic supplementary material

Supplementary Data

Supplementary Info - Full blots

## References

[1] Zhang Y (2010). Allyl isothiocyanate as a cancer chemopreventive phytochemical. Molecular Nutrition and Food Research.

[2] Srivastava SK (2003). Allyl isothiocyanate, a constituent of cruciferous vegetables, inhibits growth of PC-3 human prostate cancer xenograft *in vivo*. Carcinogenesis.

[3] Bhattacharya A (2010). Allyl isothiocyanate-rich mustard seed powder inhibits bladder cancer growth and muscle invasion. Carcinogenesis.

[4] Gupta P, Kim B, Kim SH, Srivastava SK (2014). Molecular targets of isothiocyanates in cancer: Recent advances. Mol. Nutr. Food Res..

[5] Lai KC (2014). Allyl isothiocyanate inhibits cell metastasis through suppression of the MAPK pathways in epidermal growth factor-stimulated HT29 human colorectal adenocarcinoma cells. Oncol. Rep..

[6] Tripathi K (2015). Allyl isothiocyanate induces replication-associated DNA damage response in NSCLC cells and sensitizes to ionizing radiation. Oncotarget.

[7] Xu K, Thornalley PJ (2000). Studies on the mechanism of the inhibition of human leukaemia cell growth by dietary isothiocyanates and their cysteine adducts *in vitro*. Biochem. Pharmacol..

[8] Tsai SC (2012). ERK-modulated intrinsic signaling and G2/M phase arrest contribute to the induction of apoptotic death by allyl isothiocyanate in MDA-MB-468 human breast adenocarcinoma cells. Int. J. Oncol..

[9] Sávio AL, da Silva GN, Salvadori DM (2015). Inhibition of bladder cancer cell proliferation by allyl isothiocyanate (mustard essential oil). Mutat. Res. - Fundam. Mol. Mech. Mutagen..

[10] Louhivuori LM (2009). Differentiation dependent expression of TRPA1 and TRPM8 channels in IMR-32 human neuroblastoma cells. J. Cell. Physiol..

[11] Hwang ES, Kim GH (2009). Allyl isothiocyanate influences cell adhesion, migration and metalloproteinase gene expression in SK-Hep1 cells. Exp. Biol. Med. (Maywood)..

[12] Xiao D (2003). Allyl isothiocyanate, a constituent of cruciferous vegetables, inhibits proliferation of human prostate cancer cells by causing G2/M arrest and inducing apoptosis. Carcinogenesis.

[13] Geng F (2011). Allyl isothiocyanate arrests cancer cells in mitosis, and mitotic arrest in turn leads to apoptosis via Bcl-2 protein phosphorylation. J. Biol. Chem..

[14] Lin RK (2011). Dietary isothiocyanate-induced apoptosis via thiol modification of DNA topoisomerase IIα. J. Biol. Chem..

[15] Mi L (2008). Covalent binding to tubulin by isothiocyanates. A mechanism of cell growth arrest and apoptosis. J. Biol. Chem..

[16] Thejass P, Kuttan G (2007). Allyl isothiocyanate (AITC) and phenyl isothiocyanate (PITC) inhibit tumour-specific angiogenesis by downregulating nitric oxide (NO) and tumour necrosis factor-alpha (TNF-alpha) production. Nitric Oxide - Biol. Chem..

[17] Thejass P, Kuttan G (2007). Inhibition of endothelial cell differentiation and proinflammatory cytokine production during angiogenesis by allyl isothiocyanate and phenyl isothiocyanate. Integr Cancer Ther.

[18] Higdon JV, Delage B, Williams DE, Dashwood RH (2007). Cruciferous vegetables and human cancer risk: epidemiologic evidence and mechanistic basis. Pharmacological Research.

[19] Bao Y, Wang W, Zhou Z, Sun C (2014). Benefits and Risks of the Hormetic Effects of Dietary Isothiocyanates on Cancer Prevention. PLoS One.

[20] Calabrese EJ, Baldwin LA (2002). Applications of hormesis in toxicology, risk assessment and chemotherapeutics. Trends in Pharmacological Sciences.

[21] Calabrese EJ, Iavicoli I, Calabrese V (2013). Hormesis: its impact on medicine and health. Hum. Exp. Toxicol..

[22] Son TG, Camandola S, Mattson MP (2008). Hormetic dietary phytochemicals. NeuroMolecular Medicine.

[23] Speciale A, Chirafisi J, Saija A, Cimino F (2011). Nutritional Antioxidants and Adaptive Cell Responses: An Update. Curr. Mol. Med..

[24] Pietsch K (2011). Hormetins, antioxidants and prooxidants: Defining quercetin-, caffeic acid- and rosmarinic acid-mediated life extension in C. elegans. Biogerontology.

[25] Bera D, Qian L, Tseng TK, Holloway PH (2010). Quantum dots and their multimodal applications: A review. Materials.

[26] Zrazhevskiy P, Sena M, Gao X (2010). Designing multifunctional quantum dots for bioimaging, detection, and drug delivery. Chem. Soc. Rev..

[27] Qi L, Gao X (2008). Emerging application of quantum dots for drug delivery and therapy. Expert Opin. Drug Deliv..

[28] O’Farrell N, Houlton A, Horrocks BR (2006). Silicon nanoparticles: Applications in cell biology and medicine. International Journal of Nanomedicine.

[29] Chao Y (2007). Evaporation and deposition of alkyl-capped silicon nanocrystals in ultrahigh vacuum. Nat. Nanotechnol..

[30] Cooley LS (2010). Reversible transdifferentiation of blood vascular endothelial cells to a lymphatic-like phenotype *in vitro*. J. Cell Sci..

[31] Clayton RF (2005). Liver cell lines for the study of hepatocyte functions and immunological response. Liver Int..

[32] Liu H (2013). Sulforaphane can protect lens cells against oxidative stress: Implications for cataract prevention. Investig. Ophthalmol. Vis. Sci..

[33] Collins AR (2004). The comet assay for DNA damage and repair: principles, applications, and limitations. Mol. Biotechnol..

[34] Schneider Ca, Rasband WS, Eliceiri KW (2012). NIH Image to ImageJ: 25 years of image analysis. Nat. Methods.

[35] Savio ALV, da Silva GN, Camargo EAde, Salvadori DMF (2014). Cell cycle kinetics, apoptosis rates, DNA damage and TP53 gene expression in bladder cancer cells treated with allyl isothiocyanate (mustard essential oil). Mutat. Res. - Fundam. Mol. Mech. Mutagen..

[36] Rajendran P (2013). HDAC turnover, CtIP acetylation and dysregulated DNA damage signaling in colon cancer cells treated with sulforaphane and related dietary isothiocyanates. Epigenetics.

[37] Murata M, Yamashita N, Inoue S, Kawanishi S (2000). Mechanism of oxidative DNA damage induced by carcinogenic allyl isothiocyanate. Free Radic. Biol. Med..

[38] Weis SM, Cheresh DA (2011). Tumor angiogenesis: molecular pathways and therapeutic targets. Nat. Med..

[39] Niture SK, Kaspar JW, Shen J, Jaiswal AK (2010). Nrf2 signaling and cell survival. Toxicology and Applied Pharmacology.

[40] Ahire JH (2012). Highly luminescent and nontoxic amine-capped nanoparticles from porous silicon: Synthesis and their use in biomedical imaging. ACS Appl. Mater. Interfaces.

[41] Urano Y (2009). Selective molecular imaging of viable cancer cells with pH-activatable fluorescence probes. Nat. Med..

[42] Iversen TG, Skotland T, Sandvig K (2011). Endocytosis and intracellular transport of nanoparticles: Present knowledge and need for future studies. Nano Today.

[43] Kensler TW (2013). Keap1-Nrf2 signaling: A target for cancer prevention by sulforaphane. Top. Curr. Chem..

[44] Lau A, Villeneuve NF, Sun Z, Wong PK, Zhang DD (2008). Dual roles of Nrf2 in cancer. Pharmacological Research.

[45] Moon EJ, Giaccia A (2015). Dual roles of NRF2 in tumor prevention and progression: Possible implications in cancer treatment. Free Radical Biology and Medicine.

[46] Karin M, Dhar D (2016). Liver carcinogenesis: From naughty chemicals to soothing fat and the surprising role of NRF2. Carcinogenesis.

[47] Ma X (2012). Nrf2 knockdown by shRNA inhibits tumor growth and increases efficacy of chemotherapy in cervical cancer. Cancer Chemother. Pharmacol..

[48] Fan JW (2015). Preparation, cytotoxicity and *in vivo* bioimaging of highly luminescent water-soluble silicon quantum dots. Nanotechnology.

[49] Shen P, Ohta S, Inasawa S, Yamaguchi Y (2011). Selective labeling of the endoplasmic reticulum in live cells with silicon quantum dots. Chem. Commun. (Camb)..

[50] Sakhtianchi R (2013). Exocytosis of nanoparticles from cells: Role in cellular retention and toxicity. Advances in Colloid and Interface Science.

[51] Ohta S, Inasawa S, Yamaguchi Y (2012). Real time observation and kinetic modeling of the cellular uptake and removal of silicon quantum dots. Biomaterials.

[52] Stan MS (2014). Si/SiO2 quantum dots cause cytotoxicity in lung cells through redox homeostasis imbalance. Chem. Biol. Interact..

[53] Wang AZ, Langer R, Farokhzad OC (2012). Nanoparticle Delivery of Cancer Drugs. Annu. Rev. Med..

