# Supplementary material for: Anti-cancer activities of allyl isothiocyanate and its conjugated silicon quantum dots
Source: Sci Rep. 2018 Jan 18;8:1084. doi: 10.1038/s41598-018-19353-7 (PMC5773486; doi:10.1038/s41598-018-19353-7)
Supplement: Supplementary file 1 — Supplementary Data [file 41598_2018_19353_MOESM1_ESM.docx]

Anti-cancer activities of allyl isothiocyanate and its conjugated silicon quantum dots

Peng Liu^a^, Mehrnaz Behray^b^, Qi Wang^a^, Wei Wang^a^, Zhigang Zhou^a^, Yimin Chao^b^, Yongping Bao^a^*

^a^ Norwich Medical School, University of East Anglia, Norwich, Norfolk, United Kingdom.

^b^ School of Chemistry, University of East Anglia, Norwich, Norfolk, United Kingdom.

E-Mail: [y.bao@uea.ac.uk](mailto:y.bao@uea.ac.uk)

**Supplementary Data**


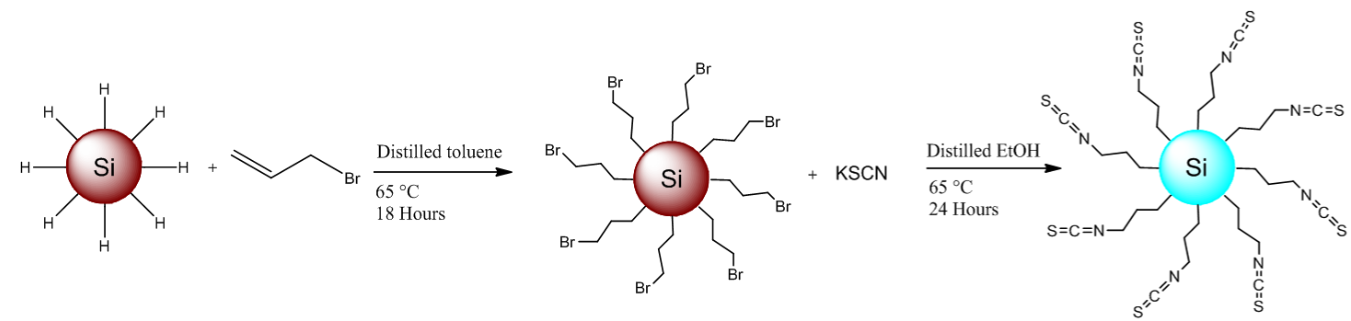


**Figure S1.** Schematic Illustration of the chemical synthesis of AITC-SiQDs.


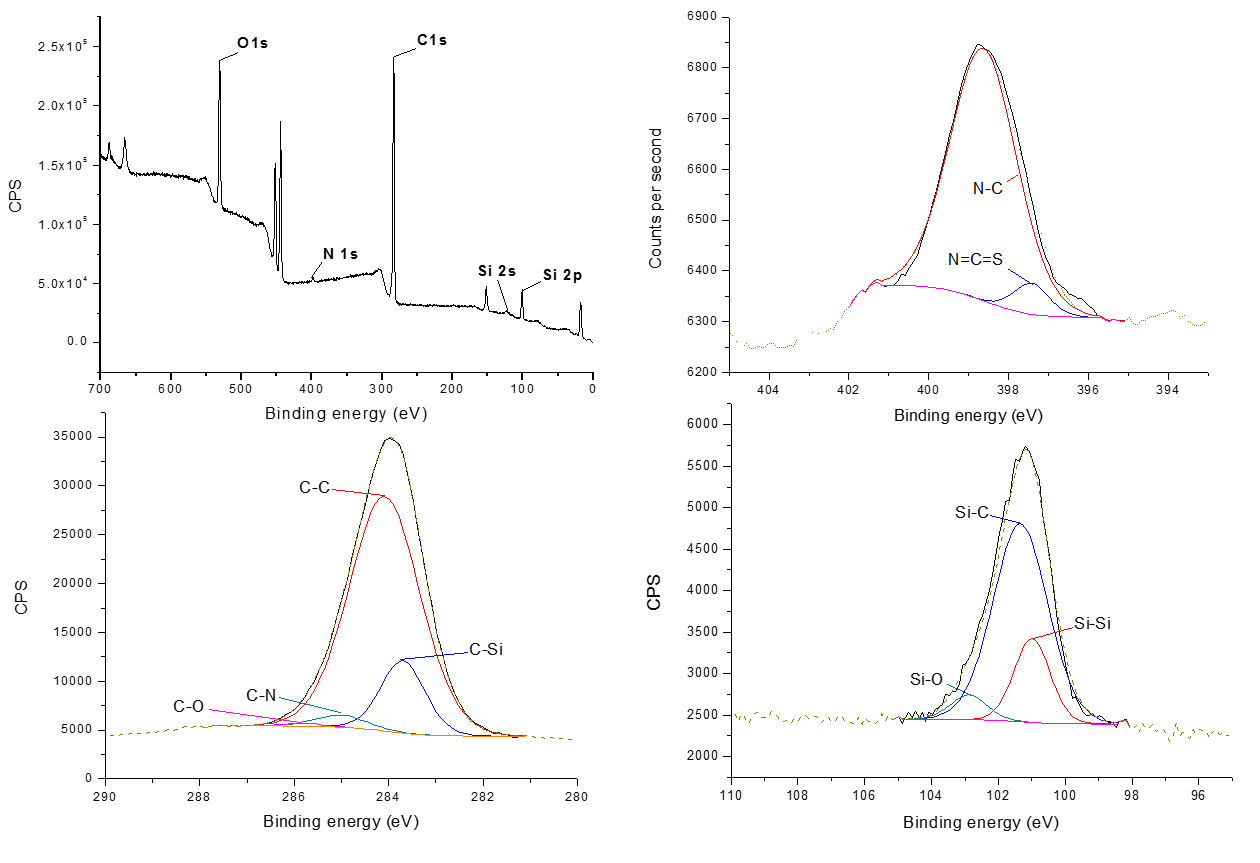


**Figure S2.** XPS Spectra obtained from AITC-SiQDs. (a) Full survey spectrum, (b) N 1s, (c) C 1s, (d) Si 2p. Dotted lines correspond to the fitting obtained by the spectrum components.


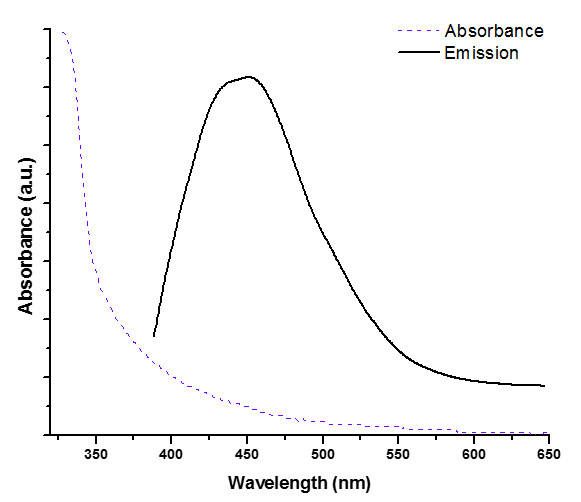


**Figure S3**. Absorption and emission spectra of AITC-SiQDs with excitation wavelength of 360 nm.


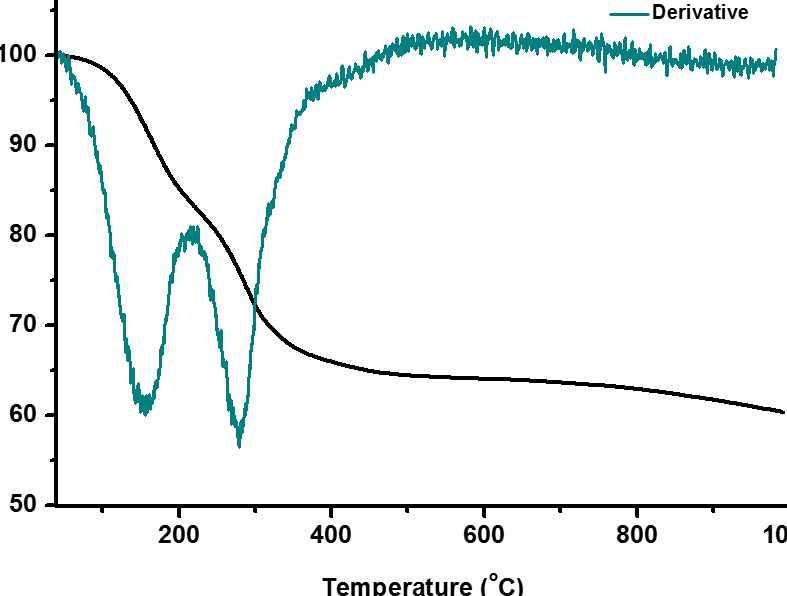


**Figure S4.** TGA and derivative of AITC-SiQDs. The analysis was performed using a METTLER-TOLEDO TGA-DSC1. The powder sample of the nanoparticles (approx. 5 mg) was placed in a 40 μL aluminium pan and measurements were taken while heating the sample between the specified temperature limits. The background measurements were performed from heating an empty pan within the same temperature range. The estimation of the weight attributed to the ligand is approximately 60% of the total nanoparticle weight, with the remaining 40% the silicon core.


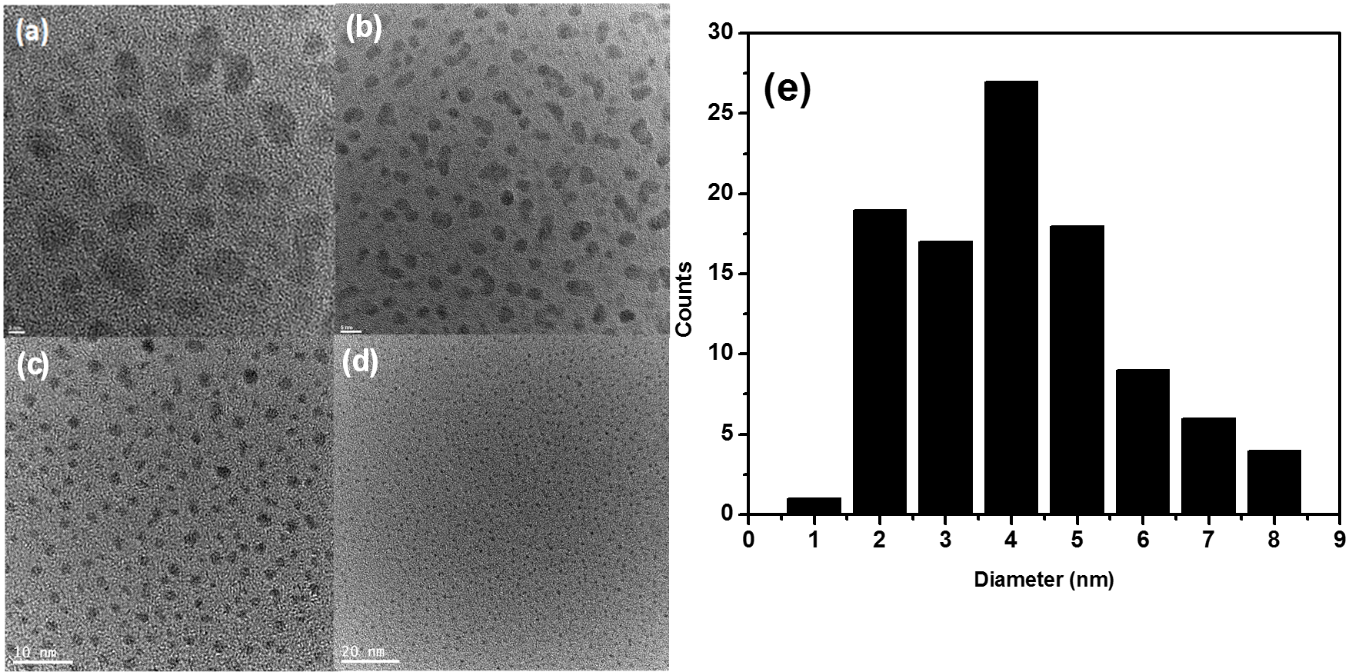


**Figure S5**. TEM images of AITC-SiQDs drop cast onto a copper grid with inset values of a) 2 nm, b) 5 nm, c) 10 nm, d) 20nm and e) corresponding histogram of sizes obtained.

**Table S1.** Hydrodynamic diameters and Polydispersity Index (PdI) of AITC-SiQDs measured by Dynamic Light Scattering (DLS) in different media.

| Solvent | Mean particle size (nm) ± SD | PdI ± SD |
| --- | --- | --- |
| Toluene | 11.85 ± 0.05 | 0.08 ± 0.02 |
| DMSO | 22.22 ± 1.20 | 0.10 ± 0.02 |
| RPMI | 22.70 ± 0.50 | 0.10 ± 0.13 |

**
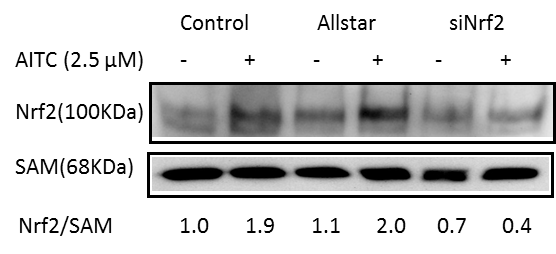
**

**Figure S6**. Effect of Nrf2 siRNA in HepG2 cells. Cells were seeded in 6 cm dishes, after 24 hours cells were treated with siNrf2 as per the manufacturer’s instructions. Allstars was used as a negative control. After another 48 hours, medium was changed with 2.5 μM AITC or DMSO (0.1%) treatment for 4 hours. Nrf2 in nuclear extract was detected using Western blot analysis.

**
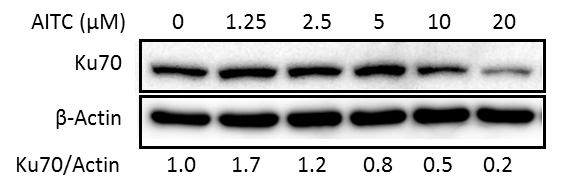
**

**Figure S7.** Effect of AITC on Ku70 total protein level in HepG2 cells. Cells were treated with different doses of AITC or DMSO (0.1%) as control for 24 hours, whole cell lysates were collected as described in Methods. Ku70 was detected by Western blot and quantified against β–Actin as a loading control, results were expressed as fold induction relative to control.


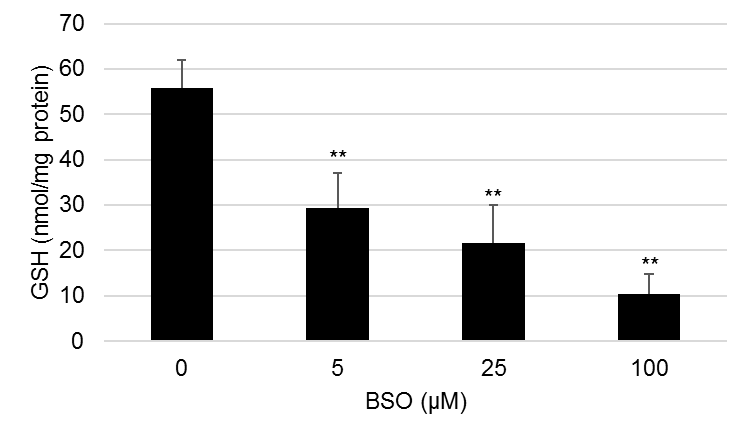


**Figure S8.** Effect of BSO on intracellular GSH levels in HepG2. Cells were incubated with different doses of BSO for 24 hours. The levels of intracellular GSH were measured by an HPLC assay (1). Data are presented as mean ± SD (n = 3), ** p< 0.01 compared to control.


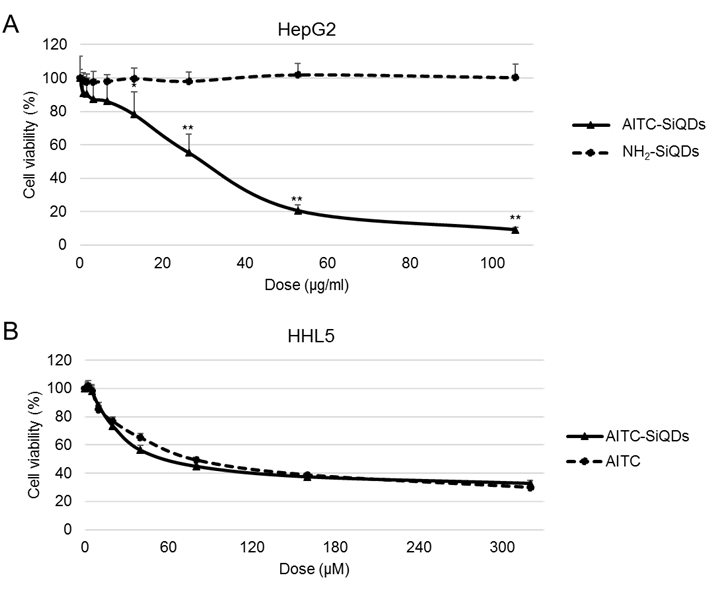


**Figure S9**. Control studies of the AITC-SiQDs cytotoxicity. (A) Effect of AITC-SiQDs was compared with NH_2_-SiQDs on HepG2 cell viability. As the ligand coverage of NH_2_-SiQDs is unknown, the unit μg/mL was used here to compare these two kinds of NPs. (B) Effect of AITC-SiQDs was compared with AITC on HHL5 cell viability. Cells were treated at 70–80% confluence for 24 hours with DMSO (0.1%) as control, and cell viability was determined by the MTT assay. Data are presented as mean ± SD (n ≥ 5), statistical significance within groups treated with the same dose, *p < 0.05, **p < 0.01.


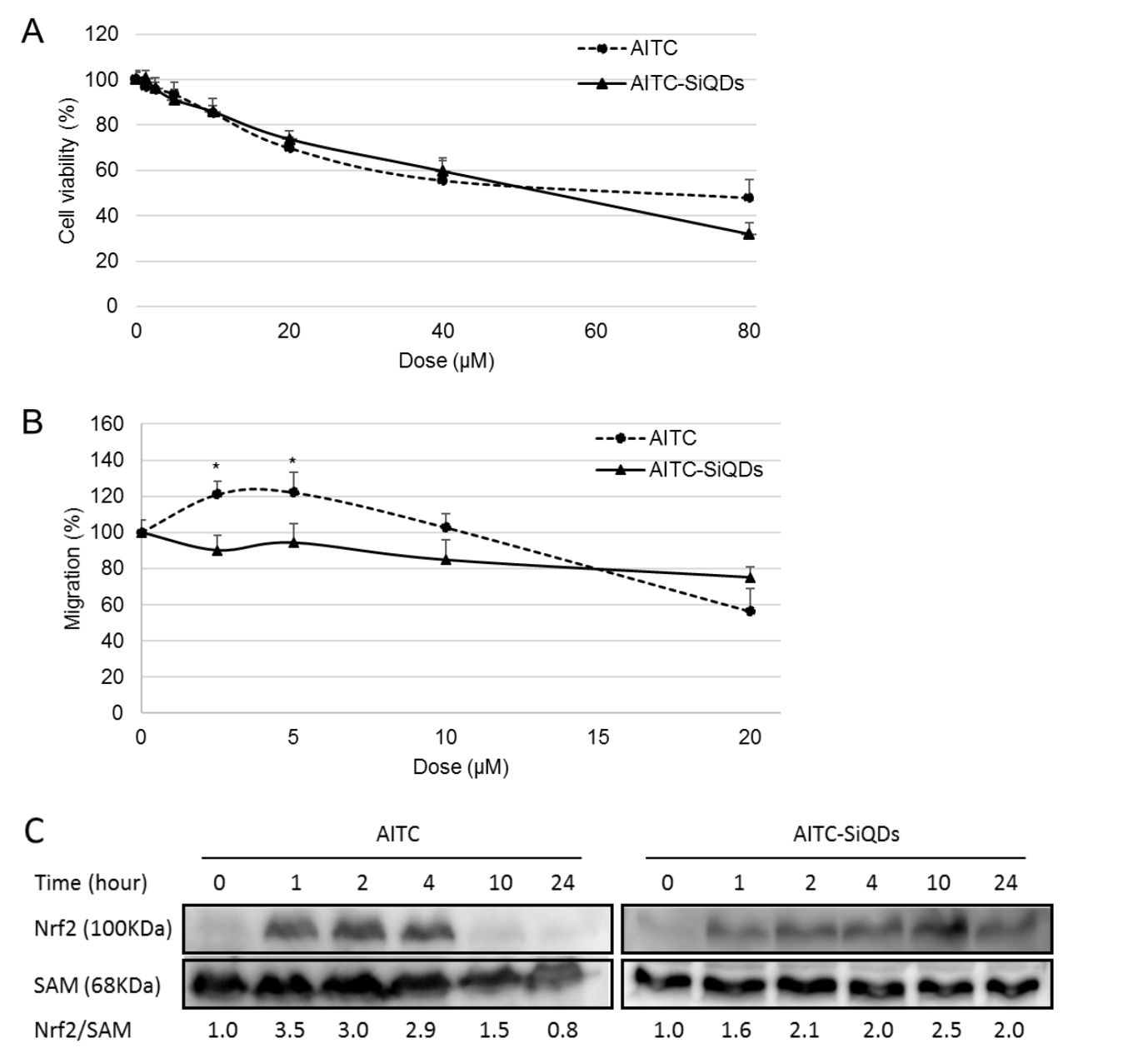


**Figure S10.** Effect of AITC or AITC-SiQDs on Caco-2 cell viability, migration and nuclear Nrf2 accumulation. (A) Cell viability at 24 hours treatment was determined by MTT assay. (B) Cell migration at 48 hours treatment was measured by wound assay. Data are presented as mean ± SD (n ≥ 5), *p < 0.05 compared to corresponding AITC treatment. (C) Time course of the effect of 20 µM AITC or AITC-SiQDs on Nrf2 nuclear protein level. Nuclear protein fractions were isolated as described in Methods. Nrf2 was detected by Western blot and quantified against SAM as a loading control.

**References**

1. Wang W, He Y, Yu G, Li B, Sexton DW, Wileman T, et al. Sulforaphane protects the liver against CdSe quantum dot-induced cytotoxicity. PLoS One. 2015;10(9).
