# Supplementary material for: Anti-cancer activities of allyl isothiocyanate and its conjugated silicon quantum dots
Source: Sci Rep. 2018 Jan 18;8:1084. doi: 10.1038/s41598-018-19353-7 (PMC5773486; doi:10.1038/s41598-018-19353-7)
Supplement: Supplementary file 2 — Supplementary Info - Full blots [file 41598_2018_19353_MOESM2_ESM.docx]

**Supplementary information - original scans for the Western blots**

Title: Anti-cancer activities of allyl isothiocyanate and its conjugated silicon quantum dots

Authors and affiliations

Peng Liu^a^, Mehrnaz Behray^b^, Qi Wang^a^, Wei Wang^a^, Zhigang Zhou^a^, Yimin Chao^b^, Yongping Bao^a,^*

^a^ Norwich Medical School, University of East Anglia, Norwich, Norfolk, United Kingdom.

^b^ School of Chemistry, University of East Anglia, Norwich, Norfolk, United Kingdom.


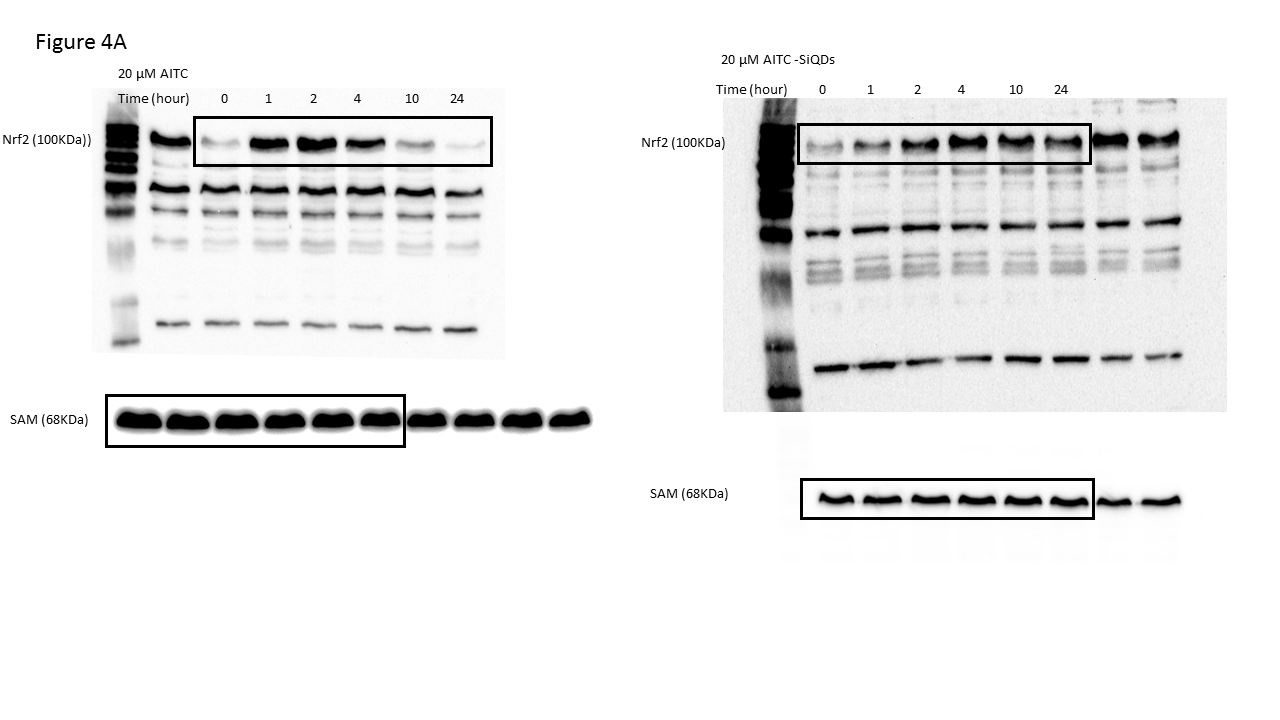


Figure 4A. Time course of the effect of 20 µM AITC or AITC-SiQDs on Nrf2 nuclear protein level.

Fig 4B.


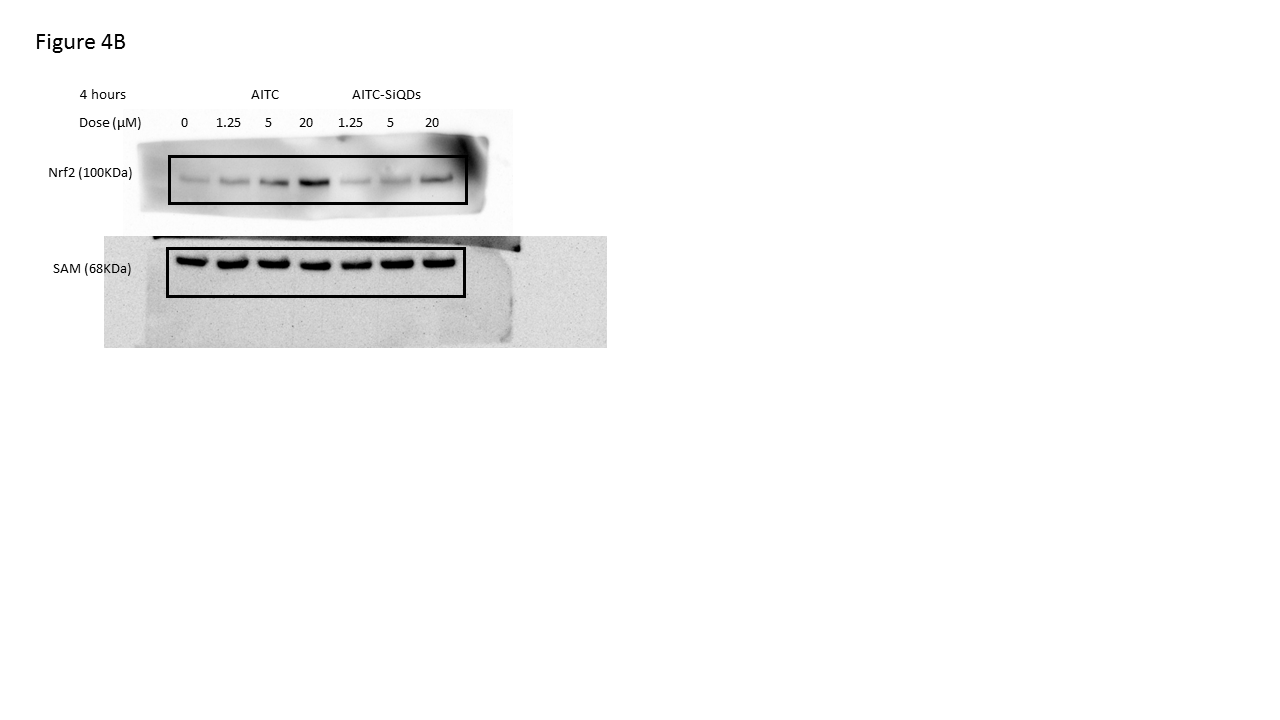


Figure 4B. Dose response of AITC or AITC-SiQDs at 4 hours on Nrf2 nuclear protein level.


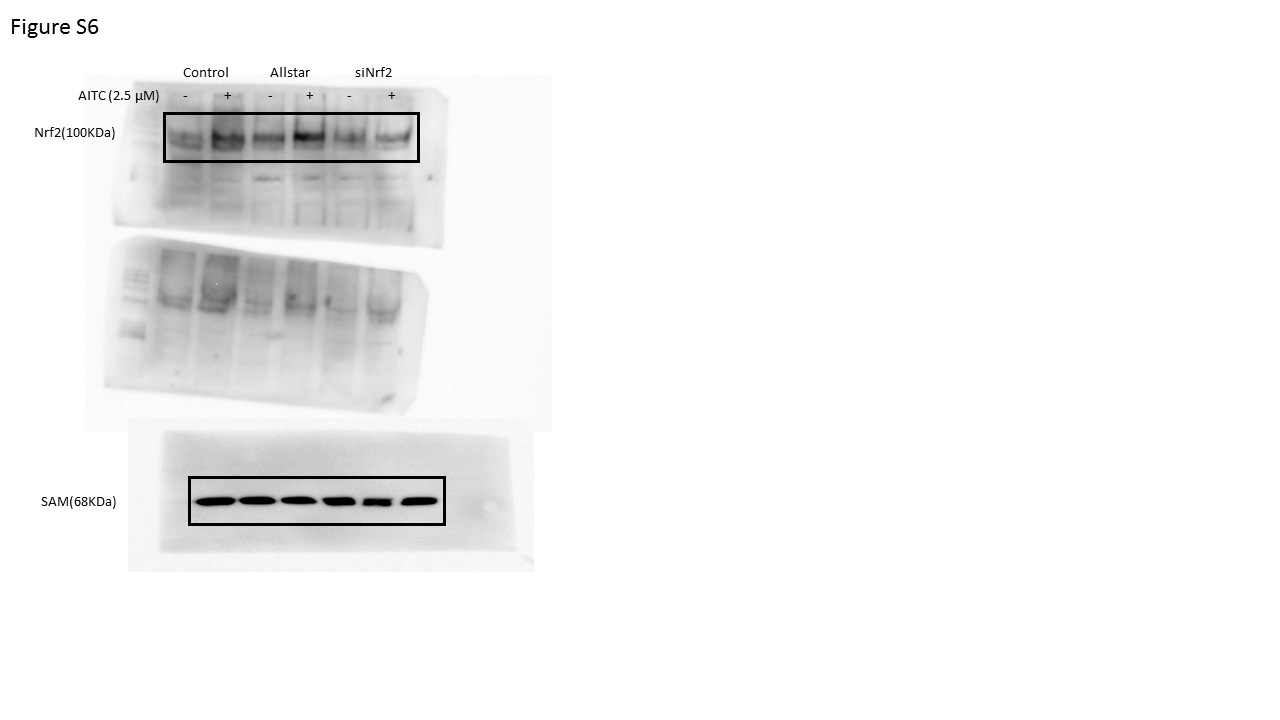


Figure S6. Effect of Nrf2 siRNA in HepG2 cells.


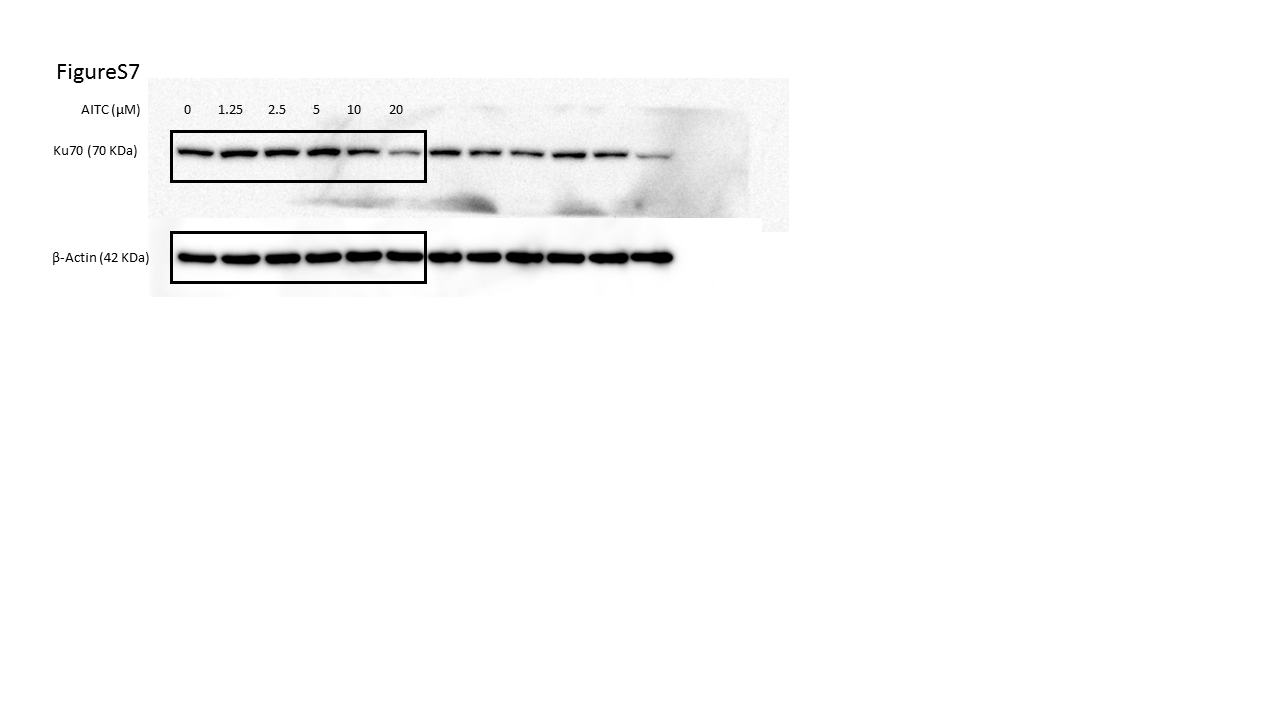


Figure S7. Effect of AITC on Ku70 protein level in HepG2 cells.


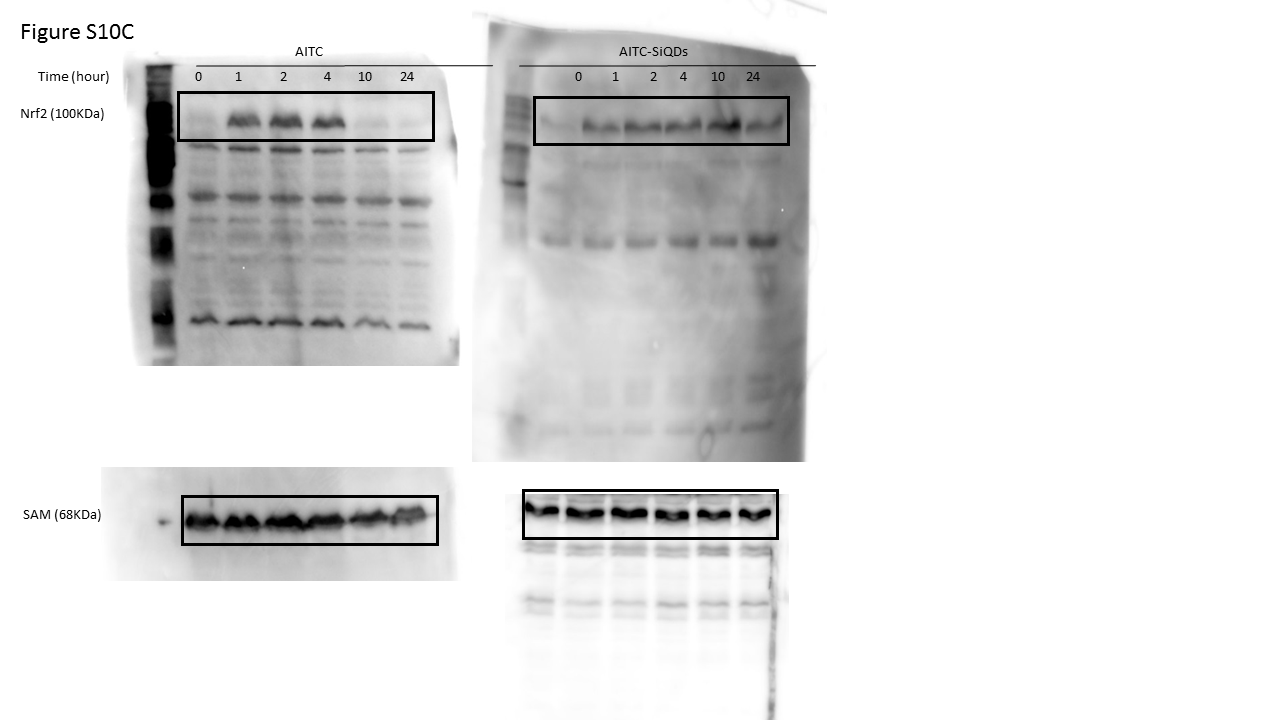


Figure S10C. Time course of the effect of 20 µM AITC or AITC SiQDs on Nrf2 nuclear protein level.
